# Functional Variant in Complement *C3* Gene Promoter and Genetic Susceptibility to Temporal Lobe Epilepsy and Febrile Seizures

**DOI:** 10.1371/journal.pone.0012740

**Published:** 2010-09-16

**Authors:** Sarah Jamali, Annick Salzmann, Nader Perroud, Magali Ponsole-Lenfant, Jennifer Cillario, Patrice Roll, Nathalie Roeckel-Trevisiol, Ariel Crespel, Jorg Balzar, Kurt Schlachter, Ursula Gruber-Sedlmayr, Ekaterina Pataraia, Christoph Baumgartner, Alexander Zimprich, Fritz Zimprich, Alain Malafosse, Pierre Szepetowski

**Affiliations:** 1 INSERM UMR 910, University of Méditerranée, Marseille, France; 2 Department of Medical Genetics and Development, University Hospital of Geneva, Geneva, Switzerland; 3 Department of Psychiatry, University Hospital of Geneva, Geneva, Switzerland; 4 Mediterranean Institute of Neurobiology (INMED), INSERM UMR901, University of Méditerranée, Marseille, France; 5 Epilepsy Unit, University Hospital of Montpellier, Montpellier, France; 6 Department of Clinical Neurology, Medical University of Vienna, Vienna, Austria; 7 Department of Pediatrics, LKH Bregenz, Bregenz, Austria; 8 Department of Pediatrics, Medical University of Graz, Austria; 9 2nd Neurological Department, General Hospital Hietzing with Neurological Center Rosenhuegel, Vienna, Austria; Instituto de Biomedicina de Valencia, CSIC, Spain

## Abstract

**Background:**

Human mesial temporal lobe epilepsies (MTLE) represent the most frequent form of partial epilepsies and are frequently preceded by febrile seizures (FS) in infancy and early childhood. Genetic associations of several complement genes including its central component *C3* with disorders of the central nervous system, and the existence of C3 dysregulation in the epilepsies and in the MTLE particularly, make it the *C3* gene a good candidate for human MTLE.

**Methodology/Principal Findings:**

A case-control association study of the *C3* gene was performed in a first series of 122 patients with MTLE and 196 controls. Four haplotypes (HAP1 to 4) comprising GF100472, a newly discovered dinucleotide repeat polymorphism [(CA)8 to (CA)15] in the *C3* promoter region showed significant association after Bonferroni correction, in the subgroup of MTLE patients having a personal history of FS (MTLE-FS+). Replication analysis in independent patients and controls confirmed that the rare HAP4 haplotype comprising the minimal length allele of GF100472 [(CA)8], protected against MTLE-FS+. A fifth haplotype (HAP5) with medium-size (CA)11 allele of GF100472 displayed four times higher frequency in controls than in the first cohort of MTLE-FS+ and showed a protective effect against FS through a high statistical significance in an independent population of 97 pure FS. Consistently, (CA)11 allele by its own protected against pure FS in a second group of 148 FS patients. Reporter gene assays showed that GF100472 significantly influenced *C3* promoter activity (the higher the number of repeats, the lower the transcriptional activity). Taken together, the consistent genetic data and the functional analysis presented here indicate that a newly-identified and functional polymorphism in the promoter of the complement *C3* gene might participate in the genetic susceptibility to human MTLE with a history of FS, and to pure FS.

**Conclusions/Significance:**

The present study provides important data suggesting for the first time the involvement of the complement system in the genetic susceptibility to epileptic seizures and to epilepsy.

## Introduction

The mammalian complement system is a key component of innate immunity and participates in adaptive immunity. It is composed of more than 30 plasma and cell surface proteins that interact in a very precise and regulated way[1]. Besides their canonical roles, there is increasing evidence that components of the complement system also participate in various physiological processes of the central nervous system (CNS) such as synapse elimination during development[2] or adult neurogenesis[3]. Complement activation may occur in several CNS pathological conditions[4], [5], including Alzheimer's disease[6] and multiple sclerosis[7]. Several proteins with complement control modules such as LEV-9[8] and SRPX2[9] are involved in acetylcholine receptor clustering at the synapse in *Caenorhabditis elegans* or in developmental disorders of the speech cortex, respectively. Polymorphisms in genes encoding several complement factors have been successfully associated with age-related macular degeneration[10]. More recently, genome-wide association studies showed involvement of the complement lysis inhibitor (*Clusterin*) gene and of the complement component receptor 1 (*CR1*) gene in the genetic risk to late onset Alzheimer's disease[11], [12].

Experimental evidences for a role of the complement system in epileptic processes have also been reported[13], [14]. Increased expression of *C3* gene and protein, and activation of the complement system have been found in brain tissues from patients with mesial temporal lobe epilepsy (MTLE)[15], [16] and from rodent MTLE model[17]. Human MTLE are the most frequent form of partial epilepsies and frequently display resistance to anti-epileptic drugs[18]. Hence MTLE represent a major health care problem. The MTLE phenotype is frequently associated with history of often-complex febrile seizures (FS) and/or with hippocampal sclerosis (HS). Familial cases with a Mendelian mode of inheritance have been reported[19], [20], [21] and a few MTLE loci have been mapped[22], [23], [24] but no gene has been identified yet. Moreover, most MTLE cases look sporadic and may be influenced by variations in multiple genes as well as by environmental factors. It is believed that the identification of susceptibility genes for MTLE will be obtained by a combination of genetic mapping and candidate gene strategies[25]. Indeed, numerous association studies have suggested the involvement of various genes in human MTLE[22] but data have mainly shown negative or conflicting[26], [27].

There is growing evidence for an important role of the complement system in CNS development and functioning and for the involvement of the complement system and particularly of C3 dysregulation, in the epilepsies – including the MTLE. Hence, the *C3* gene represented a good candidate for the genetic susceptibility to human MTLE. In the present study, we identified a novel functional, regulatory CA-repeat polymorphism (GF100472) within the promoter region of *C3* and aimed at evaluating the possibility of genetic association between human MTLE and GF100472-related haplotypes.

## Results

### Novel microsatellite repeat (GF100472) within the *C3* promoter region

**Figure 1:**
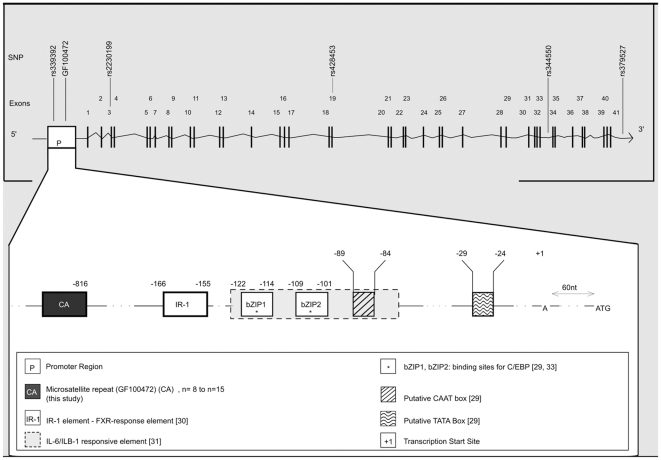
Promoter region of the human complement *C3* gene. Schematic diagram of (top) the human complement *C3* gene with exon/intron genomic structure, and (bottom) of its promoter region from the CA-repeat polymorphism marker GF100472 to the start codon. Regulatory elements and nucleotide (nt) positions respective to the canonical transcription start site (+1) are indicated.

The C3 component is encoded by the *C3* gene (Genbank NM_000064) located at chromosome 19p13. This gene extends over 42.8 kb and encompasses 41 exons (http://genome.ucsc.edu/). As a primary analysis we focused on the *C3* promoter region and on its genetic variations that may sustain modulation of *C3* transcriptional activity. The promoter region of *C3* spans more than 1 kb[28], [29]. Several *C3* regulatory elements have already been described, 5′ to the canonical transcription start site (+1) that in turn is situated 61 nucleotides (nt) upstream from the start codon (Figure 1). These include IR-1 element[30], IL6/ILβ1 responsive element[31], estrogen-response elements[32], binding sites bZIP1 and bZIP2 for transcription factor C/EBPΔ[29], [33] as well as putative CAAT and TATA boxes[29]. Careful examination of more distal 5′ sequences revealed the existence of a CA dinucleotide repeated eight times – (CA)8 – according to the reference human genome sequence at UCSC website (http://genome.ucsc.edu/) (Figure 1). Although this repeat sequence was reminiscent to classical (CA)n dinucleotide polymorphisms, it did not correspond to any previously annotated polymorphic microsatellite marker. Genotyping analysis of 196 Caucasian controls (series HI-1) revealed length polymorphisms in this (CA)n repeat, with n ranging from n = 8 to n = 15. Most frequent alleles were (CA)8 (39%), (CA)11 (30%), (CA)12 (19%), and (CA)15 (11%). Two other alleles (n = 9; n = 10) showed much lower frequencies (<1%) (Table S1). Those results were confirmed in an independent population of 255 healthy individuals (series HI-2) as well as in a third series (HI-3) of 301 additional Caucasian controls (Table S1). In addition, 54 Caucasian control trios were genotyped to assess the inheritance of this (CA)n repeat. This novel microsatellite repeat was given accession number GF100472 (database of sequence tagged sites at NCBI: http://www.ncbi.nlm.nih.gov/dbSTS/).

### Multi-markers analysis identified putative at-risk and protective haplotypes for MTLE patients with history of FS

To test for genetic association between human MTLE and the GF100472 microsatellite marker located within the C3 promoter region, a first series (MT-1) of 122 MTLE patients was collected (Table 1). Amongst the 122 patients, 57 (MTLE-FS+ patients) had a history of febrile seizures (FS) and 87 (MTLE-HS+ patients) showed hippocampal sclerosis (HS) on magnetic resonance imaging (MRI). Of note, 87.7% of the MTLE-FS+ patients also displayed HS. All patients (68 females, 54 males) as well as 196 matched controls (series HI-1) that were included in this first case-control study were European Caucasian for at least two generations. In addition to GF100472, five single nucleotide polymorphisms (SNPs) were analyzed (Figure 1). These were selected according to their respective minor allele frequencies and to their locations, owning to distinct haplotype blocks spanning C3 (International HapMap project website at: http://www.hapmap.org/). For each SNP, allele and genotype frequencies in control series HI-1 were consistent with those reported from the reference population of European descent (Population HapMap CEU at http://www.ncbi.nlm.nih.gov/SNP/) (Table S1). Single marker association analysis detected a significant difference in the distribution of CA-repeat alleles between controls and MTLE-FS+ (p = 0.036) and between controls and MTLE-HS+ (p = 0.042) (Table S2). This difference was mainly accounted by a higher frequency of allele (CA)15 in patients than in controls (Table S1). However, this association did not resist Bonferroni correction for multiple testing.

**Table 1:** Demographic and clinical characteristics of two series (MT-1, MT-2) of MTLE patients, of two series (FS-1, FS-2) of FS patients and of three series (HI-1 to 3) of control individuals.

Variables	HI-1 (N = 196)	HI-2 (N = 255)	HI-3 (N = 301)	MT-1 (N = 122)	MT-2 (N = 199)	FS-1 (N = 97)	FS-2 (N = 148)
Male (%)	109 (55.6)	144 (56.5)	171 (56.8)	54 (44.3)	81 (40.7)	54 (55.7)	81 (54.7)
Female (%)	87 (44.4)	111 (43,5)	130 (43.2)	68 (55.7)	118 (59.3)	43 (44.3)	67 (45.3)
AO, yr, mean ± SD	–	–	–	15.58±5.21	NA	3.43±1.61	NA
HS (%)	–	–	–	87 (71.9)	119 (59.8)	–	–
Personal hist. of FS (%)	0 (0)	0 (0)	0 (0)	57 (50.8)	46 (23.1)	97 (100)	148 (100)
Simple FS (%)	–	–	–	38 (66.4)	NA	83 (85.6)	67* (66.3)*
Complex FS (%)	–	–	–	19 (33.6)	NA	14 (14.4)	34* (33.7)*
Family hist. FS/epilepsy (%)	0 (0)	NA	0 (0)	29 (23.8)	49 (24.6)	19 (19.6)	NA

MTLE: Mesial temporal lobe epilepsy. HS: hippocampal sclerosis. FS: Febrile seizures. AO: age at onset. SD: standard deviation. yr: years. hist: history. NA: not available.

*: FS status (whether simple or complex) unknown in 47 patients.

The six polymorphic markers, i.e. rs339392, GF100472, rs2230199, rs428453, rs344550, and rs379527, from the 5′ to the 3′ end of the *C3* gene (Figure 1), were then included for haplotype analysis. Two six-markers haplotypes that differed for markers GF100472, rs428453, rs344550, and rs379527, T-(CA)15-C-G-C-T and T-(CA)11-C-C-G-G, showed higher (p = 0.037, p = 0.0064 and p = 0.00041) and lower (p = 0.013, p = 0.0057 and p = 0.013) frequencies, respectively, in MTLE, MTLE-HS+ and MTLE-FS+ patients. To strengthen the power of our analysis and to highlight at-risk and protective haplotypes, the analysis was then done with haplotypes formed by three-markers combinations and excluding rs339392 and rs2230199.

Four three-markers haplotypes showed significant associations after correction for multiple testing (Table S3). Interestingly, two haplotypes (HAP1 and HAP2) containing the GF100472 (CA)15 allele were associated with MTLE-FS+ (p = 0.005 and p = 0.007, respectively). Conversely, haplotype HAP3 that included the (CA)8 allele was protective against MTLE-FS+ (p = 0.003). Finally, a rare haplotype (HAP4) also comprising the (CA)8 allele was also protective (p = 0.0003). As multiple positive findings were expected and found, the false discovery rate q value was calculated to quantify the joint probability of multiple findings reflecting true associations as opposed to false positives, taking into account all comparisons performed to test the three hypotheses. The six top associations of the multiple comparisons (Table S3) were attributed a q value of 0.21 or less. While, after multiple comparison correction, it was not possible to reject all of the null hypotheses at a conventional level of statistical significance, all six of them are very unlikely to represent false positives (probability that all the six findings are false positives is 0.00009).

### Replication analysis in MTLE-FS+ patients

The highly conservative Bonferroni correction[34] as well as the results of the false discovery rate analysis indicated that at least a subset of the significant data depicted above were likely true positives. However, it remained necessary to confirm and refine our data by performing a replication study in independent case and control samples. A novel set of experiments was thus conducted in new series of 199 MTLE patients (MT-2 series) and 255 control individuals (HI-2 series). While HAP1, 2 and 3 did not show any significant difference between the novel MTLE-FS+ cases and the novel controls (data not shown), data of the protective and rare HAP4 haplotype were replicated in the novel subset of MTLE-FS+ patients with high statistical significance (p = 0.00008) (Table S3).

### Analysis in pure FS populations

MTLE-FS+ and pure FS patients may well share some susceptibility genes in common[22]. Hence, an independent population of 97 patients having pure FS (series FS-1) was studied. The (CA)11 allele showed an increased frequency in HI-1 controls (30%) as compared to FS patients (24%) (Table S1). Although this difference did not reach statistical significance, this tendency suggested a possible involvement of (CA)11 in the genetic risk for pure FS. Indeed, one (CA)11-containing haplotype (HAP5) was found to be protective with high statistical significance (p = 2.222×10^−8^) when comparing controls frequency (8.9%) to FS patients frequency (0%) (Table S3). Interestingly, HAP5 had also been found four times less frequent in MTLE-FS+ patients from series MT-1 than in HI-1 controls (Table S3). In order to assess this result, a replication study was performed in an independent set of 148 pure FS patients (FS-2 series) and in the HI-2 series of control individuals. Compared to the former FS study, a consistent albeit mirror situation was obtained. As in the first FS-1 series, HAP5 was not found in any FS patient, but data did not reach the significance threshold (Table S3). However, the sole (CA)11 marker contained within HAP5 was significantly (p = 0.007) more frequent in controls (27%, series HI-2) than in FS patients (18%, series FS-2) (Table S1). Moreover, when both FS-1 and FS-2 populations on the one hand, and both HI-1 and HI-2 control samples on the other hand, were merged as a single FS group and a single control group, respectively, data for (CA)11 remained significant (p = 0.001). No statistical difference was observed when the distribution of haplotypes was compared between patients with MLTE-FS+ and those with pure FS.

### Modulation of *C3* promoter activity by GF100472 microsatellite length polymorphism

**Figure 2:**
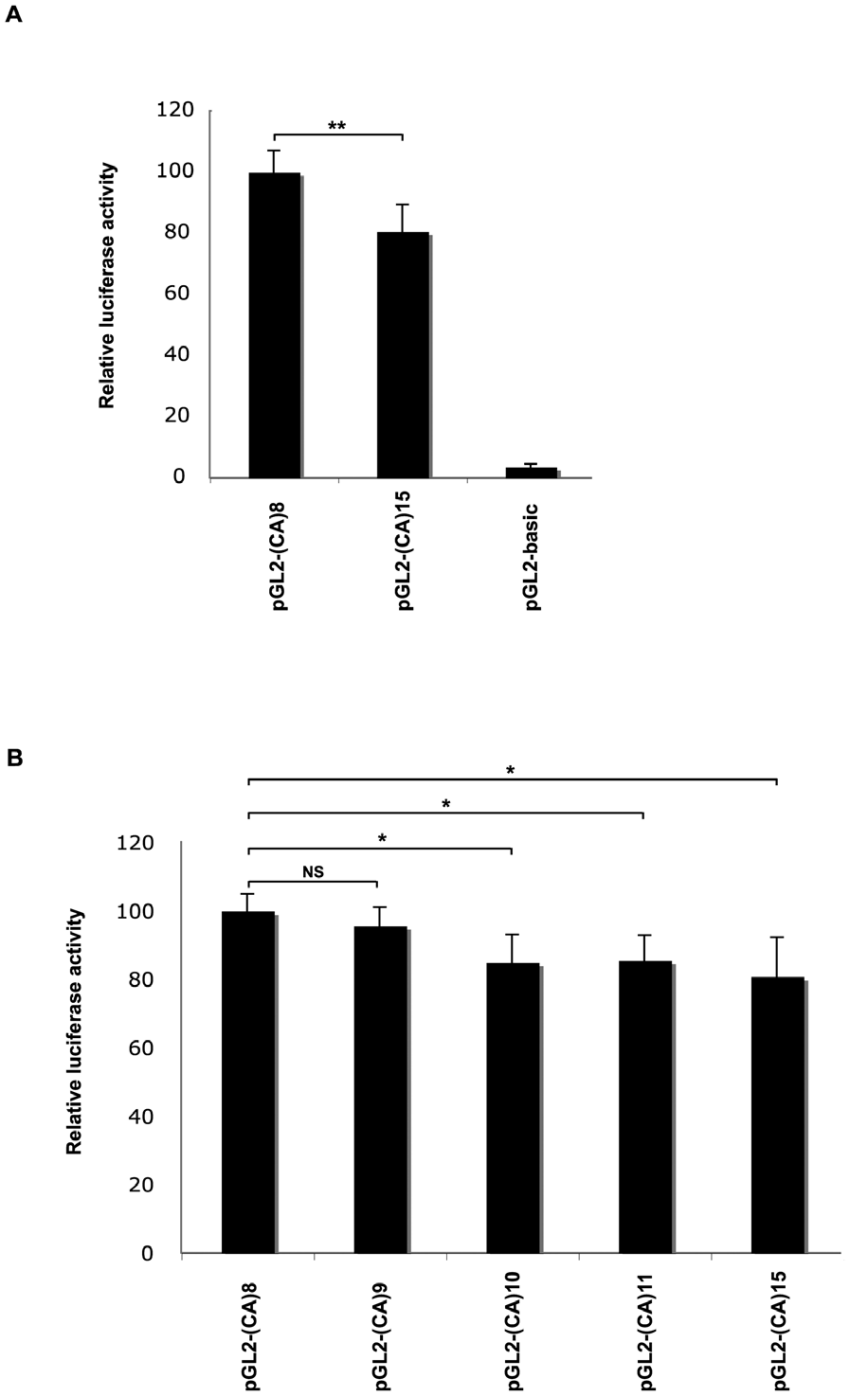
Luciferase reporter assays. (A) Comparison of the two alleles of marker GF100472 with the extreme sizes [(CA)_8_ and (CA)_15_] and (B) with either of GF100472 alleles (CA)_8_, (CA)_9_, (CA)_10_, (CA)_11_ and (CA)_15_. pGL2 vector containing the *C3* promoter region with different CA-repeat variants [(CA)_8_, (CA)_9_, (CA)_10_, (CA)_11_ and (CA)_15_] was cotransfected into HEK293T cells with a β-galactosidase expression vector (pHSV-LacZ) for normalization of transfection efficiencies. In parallel, empty (promoterless) vector (pGL2-basic) was transfected with pHSV-LacZ. Transcriptional activities were determined by quantifying the luciferase activity of cellular extracts prepared 48 h after transfection. Relative luciferase activity of (CA)_8_ was defined as 100. Data show the mean ± SD relative activity from three experiments done in triplicate. Statistical significances were determined by two-tailed unpaired *t* test. (**) indicates p<0.001. (*) indicates p<0.05. (NS) indicates non significant.

Altogether, our data indicated that the GF100472 microsatellite marker in the *C3* promoter region, influences the risk for MTLE-FS+ and for FS, by acting either on its own or at least partly in combination with other *C3* polymorphisms. Generally promoter polymorphisms might influence expression level of a gene that could account for susceptibility to develop a particular disorder. The influence on gene expression of various CA-repeats located within intronic[35] or within promoter[36], [37], [38] regions has long been described. To test for a possible effect of GF100472 on *C3* gene expression, an *in vitro* luciferase reporter gene assay was performed, by subcloning into the promoterless plasmid pGL2, several variants of the *C3* promoter regions – from nucleotides −1021 to +61 with respect to the canonical *C3* transcription start site (Figure 1). The promoter regions contained either of GF100472 alleles (CA)8, (CA)9, (CA)10, (CA)11 and (CA)15, and only differed in this number of CA-repeats, as further verified by direct full-length sequencing of each insert. Despite repeated attempts, no clone corresponding to allele (CA)12 could be obtained. Promoter constructs were transiently transfected into human embryonic kidney 293 (HEK293T) cells in order to compare their ability to drive luciferase gene expression. In a first assay, the two extreme sizes alleles [CA)8 and (CA)15] were compared. Relative levels of luciferase expression were measured in three independent experiments and indicated that the longest allele (CA)15 of GF100472 had significantly reduced promoter activity (80.69% of activity; p<0.001, unpaired t-test) as compared to the shortest allele (CA)8 (Figure 2A). All five alleles were then tested in a novel set of three independent experiments, which confirmed and extended the above data and showed that the the longer the GF100472 allele, the lower the *C3* promoter activity (Figure 2B).

## Discussion

Genetic contribution to human MTLE is well recognized but little is known about the genes participating in the underlying pathophysiology[25]. While there had been previous arguments for dysregulation of the complement system in various epileptic models and in the human MTLE particularly[15], the genetic influence of the complement system on the risk for epilepsy and seizures had not been suggested so far. In the present study, replicated genetic data and functional analysis suggest that a newly-identified and functional dinucleotide polymorphism (GF100472) in the complement component *C3* promoter influences genetic susceptibility to human MTLE with a history of FS (MTLE-FS+), and to pure FS. One rare *C3* haplotype (HAP4) that encompasses the GF100472 shortest length allele [(CA)8], protected against MTLE-FS+ in two independent populations of patients vs controls. Another rare *C3* haplotype (HAP5) was also found protective in an independent population of pure FS and was also much less frequent in MTLE-FS+ patients than in controls. Moreover, the GF100472 allele (CA)11 contained within HAP5, was also protective by itself in a replication study composed by novel series of FS patients and controls. Noteworthingly, HAP4 did not show any significant difference in an independent population of pure FS compared to matched controls. Several reasons may account for this latter data. The first one is a difference of power between the studies, hence masking some of the statistical effects. Different genetic contributions to FS developed earlier in life by patients with MTLE, and to FS displayed by the general population, might represent another and non exclusive possibility. MTLE-FS+ patients are very often considered as having complex FS[39] while FS in the general population would represent a more balanced mix of simple and complex FS. Indeed, in the present study 19 out of the 57 MTLE-FS+ patients (series MT-1) had complex FS as compared to 14 out of 97 FS patients (series FS-1) (33.6% versus 14.4%; p = 0.006). On the one hand, HAP4 could be specific to complex FS of MTLE patients and hence its effect would be diluted in the FS general population. For instance, HAP4 could influence a sequence of events leading from FS to MTLE[40] rather than directly influencing the risk of FS. On the other hand, HAP5 – including (CA)11 – might protect against FS and against MTLE as a shared susceptibility factor.

If taken separately, each association found in this study is to be considered with caution and moderate significance, both because of the limited number of patients in each MTLE and FS subgroup, and because of the scarcity of the HAP4 and HAP5 haplotypes. In such cases, there always is an inherent risk of false positive result due to chance or to bias such as occult stratification. Although this risk cannot be excluded for each separate association, the following points are noteworthy as, all taken together, they strongly argue in favor of the actual participation of the *C3* gene in the genetic susceptibility to MTLE-FS+ and to FS. First of all, the very conservative Bonferroni method was applied to correct against statistical effects of multiple testing. Moreover, the false discovery rate analysis also pointed at the existence of true positives. Secondly, protective effect of HAP4 against MTLE-FS+, while significantly displayed in samples of small sizes, was replicated in independent patients and controls groups. That rare alleles can indeed influence susceptibility to common disorders is increasingly recognized; as an example, the 15q13 microdeletion variant that was present in 1% of patients with idiopathic generalized epilepsy and that was virtually absent from controls (<0.02%), is associated with strong epileptogenic effect [41]. Thirdly, the protective effect of (CA)11-containing HAP5 haplotype in a first series of FS patients, was consistent with the protective effect of (CA)11 allele alone in a novel and independent FS population. Moreover, *C3* maps right within the 19p critical area where linkage of autosomal dominant FS trait (FEB2 locus) has been reported[42]. Fourthly, there is an obvious clinical overlap between MTLE-FS+ and FS patients and the *C3* susceptibility gene was found significantly associated in these independent populations of patients. Last but not least, the GF100472 microsatellite polymorphism included in haplotypes HAP4 and HAP5 and situated within the *C3* promoter region, influenced the promoter activity of *C3 in vitro*.

Indeed and as is always desirable in genetic association studies, we showed here that the newly discovered GF100472 microsatellite (CA-repeat) polymorphism had functional consequences. Generally, CA-repeats are purine-pyrimidine alternating sequences and hence have the potential to assume Z-DNA conformation, a left-handed double-helix structure that is thermodynamically unfavorable compared to B-DNA conformation. Z-DNA conformation has been described as negatively affecting transcriptional activity[36], [43]. Consistently, we showed that GF100472 regulated *C3* promoter activity in a length-dependent manner: the longer the CA-repeat, the lower the transcriptional activity. GF100472 may at least partly influence genetic risks to epilepsy and epileptic seizures by modulating the relative amounts of C3 transcripts. Interestingly, the IL-1β cytokine that had shown genetic association with FS and with MTLE[44] also influences *C3* promoter activity through regulatory binding elements situated in the *C3* promoter region[31], [33]. The promoter activities associated with the GF100472 alleles did not vary by more than 20%. Such a moderate effect is consistent with the participation of such alleles as susceptibility factors in multigenic disorder, as compared with the direct and more dramatic effects of mutations in monogenic disorders.

*C3* might be involved in genetic risks for human MTLE and for FS at various and non-exclusive levels and time-points. As the central component of the complement system, *C3* influences susceptibility to infectious and febrile episodes during infancy. Hence *C3* could indirectly modulate susceptibility to FS, whether simple or complex. The complement system might also play a role in the developing nervous system by tagging unwanted synapses for elimination[2]. Inter-individual variations of *C3* expression during brain development would then influence the building of neuronal circuits and the labeling of imprecise connections, hence favoring future susceptibility to seizures and epilepsy. Later in life, MTLE pathogenesis might also involve the reactivation of such developmental and complement-dependent remodeling pathways, while the complement system and C3 particularly, may also trigger neuroprotective effects during the latency period of MTLE. Overall *C3*-dependent susceptibility to FS and to MTLE-FS+ might rely on a very precise albeit evolving balance between beneficial and detrimental effects of the complement system.

## Materials and Methods

### Ethic statement

All clinical investigations were conducted according to the Declaration of Helsinki. Written informed consent was obtained from all participating subjects. The Research Ethics Board of the Department of Clinical Neurosciences of Geneva and the Medical University of Vienna reviewed and approved the study.

### Patients

Demographic and clinical characteristics of all MTLE, FS patients and all matched controls are summarized in Table 1.

The first series of 122 mesial temporal lobe epilepsy (MTLE) consisted in patients who were consecutively admitted in the Epilepsy Unit of the University Hospital of Montpellier, France. This series was designated as MT-1, while the second series (MT-2) of 199 MTLE patients was recruited at the Department of Neurology of the Medical University of Vienna, Austria (Table 1). All patients had a severe form of epilepsy with poor control of their seizures, although they were treated with two or three single anti-epileptic drugs or a combination of drugs. Diagnosis was based on patient history with a particular focus on possible history of febrile seizures (FS), clinical examination, interictal and ictal EEG analysis carried out with monitoring video-EEG, and MRI evaluation.

Patients with FS were admitted in the pediatric emergency rooms from the University Hospitals of Geneva, Lausanne and Neuchâtel (Switzerland) for the first series of 97 FS patients (series FS-1), and at Department of Pediatrics, LKH Bregenz and Department of Pediatrics, Medical University of Graz, Austria for the replication series of 148 FS patients (series FS-2). Simple FS were diagnosed when seizures were brief and lacked focal features, with a single seizure during a febrile illness. Complex FS were diagnosed if duration lasted more than 15 min, if focal features were present, or if multiple seizures occurred during a single febrile illness or within the first 24 h period.

Healthy control groups were recruited among blood donors from the University Hospital of Geneva, Switzerland (first series of 196 healthy individuals (HI), thereafter designated as series HI-1; third series of 301 controls, designated as series HI-3) and from healthy individuals in Vienna, Austria (second series of 255 controls, designated as series HI-2). To minimize morbidity among subjects in these control groups, only blood donors older than 35 years and without history of epilepsy and seizure were included.

Patients and controls were European Caucasians. Written informed consent was obtained from all participating subjects (see above).

### Genotyping

The *C3* promoter region contains a novel dinucleotide (CA) repeat polymorphism (Genbank GF100472). Oligonucleotides flanking this marker were used as primers (Table S4). PCR reaction was carried out with 100 ng of genomic DNA using Hot Star Taq DNA polymerase (Qiagen, Hilden, Germany) in a 25 µl reaction mix containing 1× buffer (Tris·Cl, KCl, (NH4)2SO4,15 mM MgCl2; pH 8.7), 0.12 mM dNTPs, 0.08 mM deaza-dNTPs, 5% DMSO, 0.4 µM primers, 1 U Taq polymerase. Amplification conditions were as follows: 95°C for 15 minutes, 30 cycles of 92°C for 1 minute, 52°C for 30 seconds and 72°C for 1 minute. For series MT-1, FS-1, HI-1 and HI-3, PCR products were analyzed by electrophoresis on a 10% polyacrylamide gel stained with ethidium bromide. For Austrian samples (series MT-2, FS-2 and HI-2) PCRs were performed with fluorescently labeled oligonucleotides and PCR products were separated on an ABI3130 Genetic Analyzer. Allele (CA)8 was 132 nucleotides (nt), allele (CA)9 was 134 nt, allele (CA)10 was 136 nt, allele (CA)11 was 138 nt, allele (CA)12 was 140 nt, allele (CA)13 was 142 nt, allele (CA)14 was 144 nt, allele (CA)15 was 146 nt. Single nucleotide polymorphisms (rs339392, rs2230199, rs428453, rs344550, and rs379527; dbSNP database at http://www.ncbi.nlm.nih.gov/SNP/) were analyzed either by direct sequencing (series MT-1, FS-1, HI-1) (ABI3700 DNA Analyzer™, Applied Biosystems) or (series MT-2, FS-2 and HI-2) by genotyping in 20 µl reaction volumes using commercially available Taq Man-based allelic discrimination assays (7700 detection system, Applied Biosystems) and standard procedures based on Applied Biosystems reagents. The list of all primers used for the corresponding PCRs is given in Table S4.

### Statistical analysis

Allele and genotype comparisons between controls and MTLE, controls and MTLE with hippocampal sclerosis (MTLE-HS+), controls and MTLE with history of FS (MTLE-FS+), and controls and FS were made using maximum likelihood inference as implanted in UNPHASED[45], which was moreover used because it allows the use of multi-allelic markers. Haplotype analysis was done following the same method where the null hypothesis is that none of the test haplotypes makes any contribution. All rare haplotypes with frequencies below 1% in both controls and patients populations were excluded from analysis, since an individual haplotype analysis would have limited statistical power. We used a sliding window procedure to extract the core haplotype associated with the trait, starting with six polymorphisms haplotype analysis and reducing the window on selected and significant haplotypes only.

Since we compared genotypic and allelic distributions of six polymorphic markers between controls and three related subgroups (MTLE, MTLE-HS+ and MTLE-FS+) in the first MTLE study (series MT-1 and HI-1), a correction for multiple testing was required. Two approaches were applied to correct for multiple non-independent comparisons. First, we used the highly conservative Bonferroni correction taking into account the non-independence of tests. We effectively tested three independent polymorphisms, since there was strong linkage disequilibrium (LD) between some of the polymorphisms (Table S5). Indeed, GF100472, rs339392 and rs2230199 were in strong LD with each others, as were rs344550 and rs379527, whereas rs428453 appeared separated from the others. Two independent tests were moreover considered for the different MTLE subgroups. As the haplotype test was a post-hoc analysis, we considered it as one additional test. Therefore, for a Bonferroni correction on the p-values, we used p = 0.05/(3×2+1)  = 0.0071 as a threshold for significance. Three unrelated samples of independent MTLE-FS+ and FS patients collected in Geneva (FS-1) and in Vienna (MT-2, FS-2), were used in the subsequent studies and the same threshold of significance was applied for the corresponding tests. Second, we applied the false discovery rate to quantify uncertainty across the multiple hypotheses tested in the six single marker tests and the multiple haplotype tests. The false discovery rate q value was therefore calculated, which denotes the expected proportion of false negatives among multiple findings. Based on the single markers and haplotypes association results, the q-value for each of these non-independent tests was calculated using the step-up procedure[46]. The q-value calculated in this way has been shown to retain desirable properties for multiple related tests in genetic association studies and can be intuitively interpreted in terms of posterior error probability.

Statistical power to detect associations was estimated using the Genetic Power Calculator (http://pngu.mgh.harvard.edu/purcell/gpc/). Thus, we determined that the case-MTLE sample in the first MTLE study had 79% power to detect a risk allele with 15% frequency and using an additive genotype model at alpha of 0.0071. The power dropped to 68% for the MTLE-HS+ and 56% for the MTLE-FS+. For the second MTLE-FS+ population and for the FS populations collected in Switzerland (FS-1) and in Austria (FS-2), powers were at 50%, 72% and 86%, respectively.

### Gene reporter assays

For luciferase gene reporter experiments, several variants of the promoter region of the *C3* gene, from nucleotides (nt) −1021 to +61 with respect to the canonical transcription start site (Figure 1), were amplified by polymerase chain reaction (PCR) from genomic DNAs of healthy donors. Primers used were as follows: PROM_C3.F: 5′– AAAAAGAGCTCGGGATGGGAGGAAGACCA; PROM_C3.R: 5′– AAAAAAGATCTGGTGCTGGGACAGTGCA. Each promoter region with either of GF100472 alleles (CA)8, (CA)9, (CA)10, (CA)11 and (CA)15, was subcloned into the pGL2 vector (Promega), 5′ to the coding sequence of the firefly luciferase reporter gene. Direct sequencing was used to confirm the integrity of the constructions and to verify that the variants only differed in the number of CA-repeats. HEK293T cells were grown in 5% CO2 at 37°C in Dulbecco's Modified Eagles Medium (DMEM) (Lonza) supplemented with 2 mM L-glutamine, 100 U/ml penicillin, 100 mg/ml streptomycin and 10% Fetal Calf Serum. One day before transfection, cells were seeded in six-well plates with a concentration of 105 cells/well. When cells reached 70–80% confluence, they were transiently cotransfected using the Lipofectamine Plus™ reagent (Invitrogen) according to the manufacturer's instructions, with 1 µg of either appropriate reporter construct (pGL2-basic for negative control, pGL2-C3 variant constructs for the analysis of each corresponding promoter activity) and with 150 ng of pHSV-LacZ vector for normalization. Forty-eight hours after transfection, cells were lysed with 150 µl of Reporter Lysis Buffer (Promega). Firefly luciferase activity was quantified using the Luciferase Assay System (Promega) on a LB9507 Luminometer (Lumat). LacZ activity was measured using the β-Galactosidase Enzyme Assay System (Promega) on a NanoDrop™ 1000 (Thermo Fisher Scientific). All transfections were performed in triplicate and repeated in three independent experiments (nine biological replicates in total) for all the variants that were subcloned. The relative luciferase activities were then calculated with correction for transfection by β-Galactosidase activity. Another set of nine replicates was also performed with the constructs corresponding to the most extreme allele sizes, i.e. (CA)8 and (CA)15. Data were expressed as mean ± SEM. Statistical significance was assessed using unpaired *t* tests (two-tailed).

## Supporting Information

Table S1 Allele and genotype frequencies in the control and patients populations for polymorphism markers used in this study.
(0.07 MB DOC)

Table S2 Single locus analysis of the *C3* gene.
(0.11 MB DOC)

Table S3 Three-locus haplotype analysis of the *C3* gene in MTLE patients and in pure FS patients.
(0.08 MB DOC)

Table S4 List of PCR primers.
(0.03 MB DOC)

Table S5 Linkage disequilibrium (LD) data.
(0.04 MB DOC)

## References

[1] Ricklin D, Lambris JD (2007). Complement-targeted therapeutics.. Nat Biotechnol.

[2] Stevens B, Allen NJ, Vazquez LE, Howell GR, Christopherson KS (2007). The classical complement cascade mediates CNS synapse elimination.. Cell.

[3] Rahpeymai Y, Hietala MA, Wilhelmsson U, Fotheringham A, Davies I (2006). Complement: a novel factor in basal and ischemia-induced neurogenesis.. EMBO J.

[4] Gasque P, Dean YD, McGreal EP, VanBeek J, Morgan BP (2000). Complement components of the innate immune system in health and disease in the CNS.. Immunopharmacology.

[5] van Beek J, Elward K, Gasque P (2003). Activation of complement in the central nervous system: roles in neurodegeneration and neuroprotection.. Ann N Y Acad Sci.

[6] Shen Y, Meri S (2003). Yin and Yang: complement activation and regulation in Alzheimer's disease.. Prog Neurobiol.

[7] Ingram G, Hakobyan S, Robertson NP, Morgan BP (2009). Complement in multiple sclerosis: its role in disease and potential as a biomarker.. Clin Exp Immunol.

[8] Gendrel M, Rapti G, Richmond JE, Bessereau JL (2009). A secreted complement-control-related protein ensures acetylcholine receptor clustering.. Nature.

[9] Roll P, Rudolf G, Pereira S, Royer B, Scheffer IE (2006). SRPX2 mutations in disorders of language cortex and cognition.. Hum Mol Genet.

[10] Jager RD, Mieler WF, Miller JW (2008). Age-related macular degeneration.. N Engl J Med.

[11] Harold D, Abraham R, Hollingworth P, Sims R, Gerrish A (2009). Genome-wide association study identifies variants at CLU and PICALM associated with Alzheimer's disease.. Nat Genet.

[12] Lambert JC, Heath S, Even G, Campion D, Sleegers K (2009). Genome-wide association study identifies variants at CLU and CR1 associated with Alzheimer's disease.. Nat Genet.

[13] Whitney KD, McNamara JO (2000). GluR3 autoantibodies destroy neural cells in a complement-dependent manner modulated by complement regulatory proteins.. J Neurosci.

[14] Xiong ZQ, Qian W, Suzuki K, McNamara JO (2003). Formation of complement membrane attack complex in mammalian cerebral cortex evokes seizures and neurodegeneration.. J Neurosci.

[15] Jamali S, Bartolomei F, Robaglia-Schlupp A, Massacrier A, Peragut JC (2006). Large-scale expression study of human mesial temporal lobe epilepsy: evidence for dysregulation of the neurotransmission and complement systems in the entorhinal cortex.. Brain.

[16] Aronica E, Boer K, van Vliet EA, Redeker S, Baayen JC (2007). Complement activation in experimental and human temporal lobe epilepsy.. Neurobiol Dis.

[17] Gorter JA, van Vliet EA, Aronica E, Breit T, Rauwerda H (2006). Potential new antiepileptogenic targets indicated by microarray analysis in a rat model for temporal lobe epilepsy.. J Neurosci.

[18] Loscher W (2002). Current status and future directions in the pharmacotherapy of epilepsy.. Trends Pharmacol Sci.

[19] Berkovic SF, McIntosh A, Howell RA, Mitchell A, Sheffield LJ (1996). Familial temporal lobe epilepsy: a common disorder identified in twins.. Ann Neurol.

[20] Cendes F, Lopes-Cendes I, Andermann E, Andermann F (1998). Familial temporal lobe epilepsy: a clinically heterogeneous syndrome.. Neurology.

[21] Depondt C, Van Paesschen W, Matthijs G, Legius E, Martens K (2002). Familial temporal lobe epilepsy with febrile seizures.. Neurology.

[22] Baulac S, Picard F, Herman A, Feingold J, Genin E (2001). Evidence for digenic inheritance in a family with both febrile convulsions and temporal lobe epilepsy implicating chromosomes 18qter and 1q25-q31.. Ann Neurol.

[23] Claes L, Audenaert D, Deprez L, Van Paesschen W, Depondt C (2004). Novel locus on chromosome 12q22-q23.3 responsible for familial temporal lobe epilepsy associated with febrile seizures.. J Med Genet.

[24] Hedera P, Blair MA, Andermann E, Andermann F, D'Agostino D (2007). Familial mesial temporal lobe epilepsy maps to chromosome 4q13.2-q21.3.. Neurology.

[25] Gambardella A, Labate A, Giallonardo A, Aguglia U (2009). Familial mesial temporal lobe epilepsies: clinical and genetic features.. Epilepsia.

[26] Tan NC, Mulley JC, Berkovic SF (2004). Genetic association studies in epilepsy: “the truth is out there”.. Epilepsia.

[27] Cavalleri GL, Weale ME, Shianna KV, Singh R, Lynch JM (2007). Multicentre search for genetic susceptibility loci in sporadic epilepsy syndrome and seizure types: a case-control study.. Lancet Neurol.

[28] Vik DP, Amiguet P, Moffat GJ, Fey M, Amiguet-Barras F (1991). Structural features of the human C3 gene: intron/exon organization, transcriptional start site, and promoter region sequence.. Biochemistry.

[29] Bruder C, Hagleitner M, Darlington G, Mohsenipour I, Wurzner R (2004). HIV-1 induces complement factor C3 synthesis in astrocytes and neurons by modulation of promoter activity.. Mol Immunol.

[30] Li J, Pircher PC, Schulman IG, Westin SK (2005). Regulation of complement C3 expression by the bile acid receptor FXR.. J Biol Chem.

[31] Wilson DR, Juan TS, Wilde MD, Fey GH, Darlington GJ (1990). A 58-base-pair region of the human C3 gene confers synergistic inducibility by interleukin-1 and interleukin-6.. Mol Cell Biol.

[32] Fan JD, Wagner BL, McDonnell DP (1996). Identification of the sequences within the human complement 3 promoter required for estrogen responsiveness provides insight into the mechanism of tamoxifen mixed agonist activity.. Mol Endocrinol.

[33] Juan TS, Wilson DR, Wilde MD, Darlington GJ (1993). Participation of the transcription factor C/EBP delta in the acute-phase regulation of the human gene for complement component C3.. Proc Natl Acad Sci U S A.

[34] Rice TK, Schork NJ, Rao DC (2008). Methods for handling multiple testing.. Adv Genet.

[35] Gebhardt F, Zanker KS, Brandt B (1999). Modulation of epidermal growth factor receptor gene transcription by a polymorphic dinucleotide repeat in intron 1.. J Biol Chem.

[36] Naylor LH, Clark EM (1990). d(TG)n.d(CA)n sequences upstream of the rat prolactin gene form Z-DNA and inhibit gene transcription.. Nucleic Acids Res.

[37] Tae HJ, Luo X, Kim KH (1994). Roles of CCAAT/enhancer-binding protein and its binding site on repression and derepression of acetyl-CoA carboxylase gene.. J Biol Chem.

[38] Yamada N, Yamaya M, Okinaga S, Nakayama K, Sekizawa K (2000). Microsatellite polymorphism in the heme oxygenase-1 gene promoter is associated with susceptibility to emphysema.. Am J Hum Genet.

[39] Wallace SJ, Arnold (2004). Febrile seizures.. Epilepsy in Children, 2nd Editions.

[40] Pitkanen A, Sutula TP (2002). Is epilepsy a progressive disorder? Prospects for new therapeutic approaches in temporal-lobe epilepsy.. Lancet Neurol.

[41] Helbig I, Mefford HC, Sharp AJ, Guipponi M, Fichera M (2009). 15q13.3 microdeletions increase risk of idiopathic generalized epilepsy.. Nat Genet.

[42] Johnson EW, Dubovsky J, Rich SS, O'Donovan CA, Orr HT (1998). Evidence for a novel gene for familial febrile convulsions, FEB2, linked to chromosome 19p in an extended family from the Midwest.. Hum Mol Genet.

[43] Delic J, Onclercq R, Moisan-Coppey M (1991). Inhibition and enhancement of eukaryotic gene expression by potential non-B DNA sequences.. Biochem Biophys Res Commun.

[44] Kauffman MA, Moron DG, Consalvo D, Bello R, Kochen S (2008). Association study between interleukin 1 beta gene and epileptic disorders: a HuGe review and meta-analysis.. Genet Med.

[45] Dudbridge F (2008). Likelihood-based association analysis for nuclear families and unrelated subjects with missing genotype data.. Hum Hered.

[46] Hochberg Y, Benjamini Y (1990). More powerful procedures for multiple significance testing.. Stat Med.

