# Supplementary material for: Functional Variant in Complement C3 Gene Promoter and Genetic Susceptibility to Temporal Lobe Epilepsy and Febrile Seizures
Source: PLoS One. 2010 Sep 16;5(9):e12740. doi: 10.1371/journal.pone.0012740 (PMC2940893; doi:10.1371/journal.pone.0012740)
Supplement: Table S1 — Allele and genotype frequencies in the control and patients populations for polymorphism markers used in this study. (0.07 MB DOC) [file pone.0012740.s001.doc]

**Table S1.** Allele and genotype frequencies in the control and patients populations for polymorphism markers used in this study. Population HapMap CEU: reference population of European descent as at the SNP database (http://www.ncbi.nlm.nih.gov/SNP/). HI-1, HI-2 and HI-3: first, second and third series of controls, respectively. FS: febrile seizures. HS: hippocampal sclerosis. MT-1, MT-2: first and second series of patients with mesial temporal lobe epilepsy (MTLE), respectively. FS-1, FS-2: first and second series of pure FS patients, respectively. .NA: not available.*: in the replication analysis of the pure FS study, the (CA)_11_ allele was significantly (p = 0.007) more frequent in controls (27%, series HI-2) than in FS patients (18%, series FS-2).

| **rs339392** | **G/G** | **G/T** | **T/T** | **G** | **T** |  |  |  |
| --- | --- | --- | --- | --- | --- | --- | --- | --- |
| HapMap | 0.07 | 0.40 | 0.53 | 0.27 | 0.73 |  |  |  |
| HI-1 | 0.03 | 0.29 | 0.68 | 0.18 | 0.82 |  |  |  |
| **GF100472** | **(CA)_8_** | **(CA)_9_** | **(CA)_10_** | **(CA)_11_** | **(CA)_12_** | **(CA)_13_** | **(CA)_14_** | **(CA)_15_** |
| HapMap | NA | NA | NA | NA | NA | NA | NA | NA |
| HI-1 | 0.39 | 0.003 | 0.008 | 0.30 | 0.19 | 0.00 | 0.00 | 0.11 |
| HI-2 | 0.39 | 0.00 | 0.003 | **0.27*** | 0.21 | 0.00 | 0.00 | 0.13 |
| HI-3 | 0.36 | 0.008 | 0.003 | 0.27 | 0.23 | 0.00 | 0.00 | 0.13 |
| MT-1 | 0.35 | 0.00 | 0.00 | 0.28 | 0.22 | 0.004 | 0.00 | 0.15 |
| MT-1 HS+ | 0.34 | 0.00 | 0.00 | 0.25 | 0.22 | 0.006 | 0.00 | 0.18 |
| MT-1 FS+ | 0.31 | 0.00 | 0.00 | 0.25 | 0.23 | 0.009 | 0.00 | 0.20 |
| MT-2 | 0.44 | 0.002 | 0.00 | 0.25 | 0.20 | 0.00 | 0.002 | 0.10 |
| MT-2 FS+ | 0.41 | 0.00 | 0.00 | 0.33 | 0.17 | 0.00 | 0.00 | 0.09 |
| FS-1 | 0.43 | 0.00 | 0.00 | 0.24 | 0.21 | 0.02 | 0.00 | 0.10 |
| FS-2 | 0.41 | 0.00 | 0.008 | **0.18*** | 0.26 | 0.004 | 0.004 | 0.13 |
| **rs2230199** | **C/C** | **C/G** | **G/G** | **C** | **G** |  |  |  |
| HapMap | 0.70 | 0.25 | 0.05 | 0.83 | 0.17 |  |  |  |
| HI-1 | 0.68 | 0.28 | 0.04 | 0.82 | 0.18 |  |  |  |
| **rs428453** | **C/C** | **C/G** | **G/G** | **C** | **G** |  |  |  |
| HapMap | 0.14 | 0.50 | 0.36 | 0.39 | 0.61 |  |  |  |
| HI-1 | 0.14 | 0.49 | 0.37 | 0.39 | 0.61 |  |  |  |
| HI-2 | 0.16 | 0.40 | 0.44 | 0.36 | 0.64 |  |  |  |
| **rs344550** | **C/C** | **C/G** | **G/G** | **C** | **G** |  |  |  |
| HapMap | 0.43 | 0.43 | 0.13 | 0.65 | 0.35 |  |  |  |
| HI-1 | 0.48 | 0.43 | 0.09 | 0.69 | 0.31 |  |  |  |
| HI-2 | 0.44 | 0.44 | 0.12 | 0.66 | 0.34 |  |  |  |
| **rs379527** | **G/G** | **G/T** | **T/T** | **G** | **T** |  |  |  |
| HapMap | 0.10 | 0.52 | 0.38 | 0.36 | 0.64 |  |  |  |
| HI-1 | 0.05 | 0.47 | 0.48 | 0.29 | 0.71 |  |  |  |
| HI-2 | 0.07 | 0.41 | 0.52 | 0.28 | 0.72 |  |  |  |
