# Supplementary material for: Functional Variant in Complement C3 Gene Promoter and Genetic Susceptibility to Temporal Lobe Epilepsy and Febrile Seizures
Source: PLoS One. 2010 Sep 16;5(9):e12740. doi: 10.1371/journal.pone.0012740 (PMC2940893; doi:10.1371/journal.pone.0012740)
Supplement: Table S2 — Single locus analysis of the C3 gene. (0.11 MB DOC) [file pone.0012740.s002.doc]

**Table S2**. Single locus analysis of the *C3* gene.

M: first series (MT-1) of patients with mesial temporal lobe epilepsies (MTLE). MH: MTLE patients of series MT-1 with hippocampal sclerosis. MF: MTLE patients of series MT-1 with personal history of febrile seizures. HI-1: healthy individuals (first series).

|  |  | HI-1 | M | MH | MF | Likelihood ratio test | | |
| --- | --- | --- | --- | --- | --- | --- | --- | --- |
|  |  | n  (%) | n  (%) | n  (%) | n  (%) | M vs  HI-1 | MH vs  HI-1 | MF vs  HI-1 |
| GF100472 | Genotypes | 196 | 122 | 87 | 57 | p | p | p |
|  | (CA)_8_-  (CA)_8_ | 28  (14.3) | 14  (11.5) | 11  (12.6) | 5  (8.7) | 0.57 | 0.177 | 0.128 |
|  | (CA)_8_-  (CA)_10_ | 1  (0.5) | 0  (0) | 0  (0) | 0  (0) |  |  |  |
|  | (CA)_8_-  (CA)_11_ | 50  (25.5) | 25  (20.5) | 14  (16.1) | 7  (12.3) |  |  |  |
|  | (CA)_8_-  (CA)_12_ | 31  (15.8) | 21  (17.2) | 12  (13.8) | 11  (19.3) |  |  |  |
|  | (CA)_8_-  (CA)_15_ | 15  (7.6) | 12  (9.8) | 11  (12.6) | 7  (12.3) |  |  |  |
|  | (CA)_9_-  (CA)_11_ | 1  (0.5) | 0  (0) | 0  (0) | 0  (0) |  |  |  |
|  | (CA)_10_-  (CA)_10_ | 1  (0.5) | 0  (0) | 0  (0) | 0  (0) |  |  |  |
|  | (CA)_11_-  (CA)_11_ | 20  (10.2) | 8  (6.6) | 4  (4.6) | 4  (7) |  |  |  |
|  | (CA)_11_-  (CA)_12_ | 15  (7.6) | 14  (11.5) | 12  (13.8) | 6  (10.5) |  |  |  |
|  | (CA)_11_-  (CA)_13_ | 0  (0) | 1  (0.8) | 1  (1.1) | 1  (1.7) |  |  |  |
|  | (CA)_11_-  (CA)_15_ | 13  (6.6) | 12  (9.8) | 9  (10.3) | 7  (12.3) |  |  |  |
|  | (CA)_12_-  (CA)_12_ | 9  (4.6) | 7  (5.7) | 6  (6.9) | 4  (7) |  |  |  |
|  | (CA)_12_-  (CA)_15_ | 9  (4.6) | 4  (3.3) | 3  (3.4) | 1  (1.7) |  |  |  |
|  | (CA)_15_-  (CA)_15_ | 3  (1.5) | 4  (3.3) | 4  (4.6) | 4  (7) |  |  |  |
|  | Alleles | 392 | 244 | 174 | 114 |  |  |  |
|  | (CA)_8_ | 153  (39) | 86  (35.3) | 59  (33.9) | 35  (30.7) | 0.17 | 0.057 | 0.036 |
|  | (CA)_9_ | 1  (0.3) | 0  (0) | 0  (0) | 0  (0) |  |  |  |
|  | (CA)_10_ | 3  (0.8) | 0  (0) | 0  (0) | 0  (0) |  |  |  |
|  | (CA)_11_ | 119  (30.4) | 68  (27.9) | 44  (25.3) | 29  (25.4) |  |  |  |
|  | (CA)_12_ | 73  (18.6) | 53  (21.7) | 39  (22.4) | 26  (22.8) |  |  |  |
|  | (CA)_13_ | 0  (0) | 1  (0.4) | 1  (0.6) | 1  (0.9) |  |  |  |
|  | (CA)_15_ | 43  (11) | 36  (14.8) | 31  (17.8) | 23  (20.2) |  |  |  |
| rs339392 | Genotypes | 184 | 117 | 85 | 55 |  |  |  |
|  | GG | 6  (3.26) | 3  (2.56) | 3  (3.53) | 1  (1.82) | 0.599 | 0.546 | 0.856 |
|  | GT | 53  (28.80) | 40  (34.19) | 30  (35.29) | 16  (29.09) |  |  |  |
|  | TT | 125  (67.93) | 74  (63.25) | 52  (61.18) | 38  (69.09) |  |  |  |
|  | Alleles | 368 | 234 | 170 | 110 |  |  |  |
|  | G | 65  (17.66) | 46  (19.66) | 36  (21.18) | 18  (16.36) | 0.538 | 0.332 | 0.752 |
|  | T | 303  (82.34) | 188  (80.34) | 134  (78.82) | 92  (83.64) |  |  |  |
| rs2230199 | Genotypes | 189 | 117 | 85 | 55 |  |  |  |
|  | CC | 128  (67.72) | 77  (65.81) | 58  (68.24) | 37  (67.27) | 0.841 | 0.974 | 0.927 |
|  | CG | 53  (28.04) | 36  (30.77) | 23  (27.06) | 15  (27.27) |  |  |  |
|  | GG | 8  (4.23) | 4  (3.42) | 4  (4.71) | 3  (5.45) |  |  |  |
|  | Alleles | 378 | 234 | 170 | 110 |  |  |  |
|  | C | 309  (81.75) | 190  (81.20) | 139  (81.76) | 89  (80.90) | 0.944 | 0.81 | 0.971 |
|  | G | 69  (18.25) | 44  (18.80) | 31  (18.23) | 21  (19.10) |  |  |  |
| rs428453 | Genotypes | 186 | 119 | 86 | 56 |  |  |  |
|  | CC | 27  (14.52) | 13  (10.92) | 9  (10.47) | 6  (10.71) | 0.642 | 0.608 | 0.722 |
|  | CG | 91  (48.92) | 59  (49.58) | 42  (48.84) | 30  (27.76) |  |  |  |
|  | GG | 68  (36.56) | 47  (39.50) | 35  (40.70) | 20  (35.71) |  |  |  |
|  | Alleles | 372 | 238 | 172 | 112 |  |  |  |
|  | C | 145  (38.98) | 85  (35.71) | 60  (34.88) | 42  (37.50) | 0.417 | 0.359 | 0.778 |
|  | G | 227  (61.02) | 153  (64.29) | 112  (65.12) | 70  (62.50) |  |  |  |
| rs344550 | Genotypes | 190 | 116 | 84 | 56 |  |  |  |
|  | CC | 91  (47.89) | 52  (44.83) | 36  (42.86) | 26  (46.43) | 0.736 | 0.74 | 0.582 |
|  | CG | 81  (42.63) | 50  (43.10) | 39  (46.43) | 22  (39.29) |  |  |  |
|  | GG | 18  (9.47) | 14  (12.07) | 9  (10.71) | 8  (14.29) |  |  |  |
|  | Alleles | 380 | 232 | 168 | 112 |  |  |  |
|  | C | 263  (69.21) | 154  (66.38) | 111  (66.07) | 74  (66.07) | 0.466 | 0.467 | 0.53 |
|  | G | 117  (30.79) | 78  (33.62) | 57  (33.93) | 38  (33.93) |  |  |  |
| rs379527 | Genotypes | 180 | 117 | 85 | 56 |  |  |  |
|  | GG | 9  (5.00) | 13  (11.11) | 9  (10.59) | 7  (12.50) | 0.133 | 0.22 | 0.126 |
|  | GT | 85  (47.22) | 49  (41.88) | 40  (47.06) | 22  (39.29) |  |  |  |
|  | TT | 86  (47.78) | 55  (47.01) | 36  (42.35) | 27  (48.21) |  |  |  |
|  | Alleles | 360 | 234 | 170 | 112 |  |  |  |
|  | G | 103  (28.61) | 75  (32.05) | 58  (34.12) | 36  (32.14) | 0.371 | 0.198 | 0.474 |
|  | T | 257  (71.39) | 159  (67.95) | 112  (65.88) | 76  (67.86) |  |  |  |
