# Supplementary material for: Functional Variant in Complement C3 Gene Promoter and Genetic Susceptibility to Temporal Lobe Epilepsy and Febrile Seizures
Source: PLoS One. 2010 Sep 16;5(9):e12740. doi: 10.1371/journal.pone.0012740 (PMC2940893; doi:10.1371/journal.pone.0012740)
Supplement: Table S3 — Three-locus haplotype analysis of the C3 gene in MTLE patients and in pure FS patients. (0.08 MB DOC) [file pone.0012740.s003.doc]

**Table S3**. Three-locus haplotype analysis of the *C3* gene in MTLE patients and in pure FS patients.

| **GF100472**  **allele** | **rs**  **428453**  **allele** | **rs**  **344550**  **allele** | **rs**  **379527**  **allele** | **C** | **M** | **MH** | **MF** | **F** | **M vs C** | | **MH vs C** | | **MF vs C** | | **F vs C** | |
| --- | --- | --- | --- | --- | --- | --- | --- | --- | --- | --- | --- | --- | --- | --- | --- | --- |
|  |  |  |  | **Fr** | **Fr** | **Fr** | **Fr** | **Fr** | **p value** | **OR**  **(95%CI)** | **p value** | **OR**  **(95%CI)** | **p value** | **OR**  **(95%CI)** | **p value** | **OR**  **(95%CI)** |
| ***Series 1*** |  |  |  |  |  |  |  |  |  |  |  |  |  |  |  |  |
| (CA)_8_ | G | G | – | 0.093 | 0.088 | 0.073 | 0.020 | 0.119 | 0.781 | 0.56  (0.15–2.06) | 0.442 | 0.43  (0.11–1.75) | 0.020 | 0.11  (0.1–0.98) | 0.768 | 2.65  (0.25–27.93) |
| (CA)_15_  **HAP1** | G | C | – | 0.038 | 0.079 | 0.113 | 0.121 | 0.52 | 0.047 | 1.23  (1.05–5.87) | **0.005** | 1.64  (1.34–7.92) | **0.005** | 1.59  (1.31–8.04) | 0.498 | 2.89  (0.17–47.75) |
| (CA)_11_  HAP5 | C | – | G | 0.089 | 0.022 | 0.020 | 0.021 | 0 | 0.046 | 0.09  (0.08–0.53) | 0.054 | 0.09  (0.1–1.43) | 0.102 | 0.08  (0.07–1.42) | **2.222 e^–8^** | NA |
| (CA)_8_  **HAP3** | G | – | G | 0.093 | 0.072 | 0.062 | 0.014 | 0.078 | 0.404 | 0.29  (0.1–2.67) | 0.309 | 0.24  (0.1–2.56) | **0.003** | 0.5  (0.1–0.97) | 0.402 | 0.26  (0.02–3.98) |
| (CA)_12_ | G | – | G | 0.041 | 0.076 | 0.097 | 0.118 | 0.020 | 0.197 | 1.68  (0.1–6.63) | 0.042 | 1.83  (1.08–8.21) | 0.083 | 1.97  (0.1–6.71) | 0.899 | 0.15  (0.03–6.43) |
| (CA)_15_  HAP2 | G | – | T | 0.023 | 0.054 | 0.078 | 0.092 | 0.053 | 0.149 | 1.84  (0.82–8.66) | 0.009 | 1.18  (1.11–12.37) | **0.007** | 1.31  (1.12–10.14) | 0.362 | 0.713  (0.04–12.89) |
| (CA)_8_  **HAP4** | – | G | T | 0.025 | 0 | 0 | 0 | 0.047 | 0.28 | NA | – | – | **0.0003** | NA | 0.453 | 0.32  (0.001–13.5) |
| (CA)_15_ | – | C | T | 0.037 | 0.077 | 0.099 | 0.101 | 0.073 | 0.157 | 3.29  (0.17–62.64) | 0.019 | 2.95  (1.55–56.23) | 0.041 | 2.05  (1.04–40.4) | 0.261 | 0.35  (0.009–13.29) |
| (CA)_12_ | – | G | G | 0.038 | 0.096 | 0.112 | 0.136 | 0.092 | 0.166 | 3.88  (0.19–77.33) | 0.087 | 3.19  (0.16–64.07) | 0.027 | 2.64  (1.31–53.43) | 0.356 | 0.42  (0.10 –16.21) |
| ***Series 2*** |  |  |  |  |  |  |  |  |  |  |  |  |  |  |  |  |
| (CA)_8_ | G | G | – | 0.072 | 0.090 | – | 0.062 | 0.107 | 0.534 | 1.17  (0.42–3.22) | – | – | 0.434 | 0.79  (0.15–4.32) | 0.693 | 0.92 (0.32–2.62) |
| (CA)_15_  HAP1 | G | C | – | 0.066 | 0.052 | – | 0.035 | 0.094 | 0.455 | 0.73  (0.25–2.13) | **–** | – | 0.458 | 0.49  (0.08–2.91) | 0.506 | 0.88  (0.30–2.58) |
| (CA)_11_  HAP5 | C | – | G | 0.011 | 0.021 | – | 0 | 0 | 0.751 | 2.14  (0.21–21.47) | – | – | 0.03 | NA | 0.662 | NA |
| (CA)_8_  HAP3 | G | – | G | 0.052 | 0.096 | – | 0.093 | 0.109 | 0.185 | 2.13  (0.60–7.53) | – | – | 0.998 | 4.84  (0.30–78.88) | 0.355 | 4.17  (0.89–19.57) |
| (CA)_12_ | G | – | G | 0.065 | 0.057 | – | 0.063 | 0.062 | 0.489 | 1.02  (0.35–3.00) | – | – | 0.287 | 2.64  (0.25–27.81) | 0.205 | 1.90  (0.50–7.18) |
| (CA)_15_  HAP2 | G | – | T | 0.064 | 0.055 | – | 0.040 | 0.092 | 0.408 | 0.98  (0.35–2.78) | – | – | 0.379 | 1.72  (0.12–24.96) | 0.704 | 2.87  (0.82–9.97) |
| (CA)_8_  **HAP4** | – | G | T | 0.022 | 0.013 | – | 0 | 0.038 | 0.41 | 0.53  (0.12–2.31) | – | – | **0.00008** | NA | 0.304 | NA |
| (CA)_15_ | – | C | T | 0.086 | 0.061 | – | 0.100 | 0.081 | 0.324 | 0.64  (0.32–1.28) | – | – | 0.019 | 2.05  (1.09–46.97) | 0.933 | NA |
| (CA)_12_ | – | G | G | 0.073 | 0.061 | – | 0.136 | 0.097 | 0.434 | 0.75  (0.35–1.62) | – | – | 0.028 | 2.64  (1.12–58.29) | 0.344 | NA |

Results were obtained by comparing (top) the first series (series 1) of 122 MTLE (MT-1, mesial temporal lobe epilepsies; M), 87 MTLE-HS+ (MTLE with hippocampal sclerosis; MH) and 57 MTLE-FS+ (MTLE with personal history of febrile seizures; MF) patients, and the first series of 97 pure FS (FS-1; F) patients with the first series (HI-1) of 196 controls (C), and (bottom) the second series (series 2) of 199 MTLE (MT-2, mesial temporal lobe epilepsies; M) and 46 MTLE-FS+ (MTLE with personal history of febrile seizures; MF) patients, and the second series of 148 pure FS (FS-2; F) patients with the second series (HI-2) of 255 control individuals (C). The three-locus haplotypes (HAP1–5) that are specifically mentioned in the core text of the manuscript are also indicated in the first column of the table. Fr: frequency. OR: odds ratio. CI: confidence interval. p values and haplotypes that remained significant after Bonferroni’s correction are in boldface. NA: non-applicable due to absence of HAP4 in the patients. –: Not relevant or not done.
