# Supplementary material for: Functional Variant in Complement C3 Gene Promoter and Genetic Susceptibility to Temporal Lobe Epilepsy and Febrile Seizures
Source: PLoS One. 2010 Sep 16;5(9):e12740. doi: 10.1371/journal.pone.0012740 (PMC2940893; doi:10.1371/journal.pone.0012740)
Supplement: Table S4 — List of PCR primers. (0.03 MB DOC) [file pone.0012740.s004.doc]

**Table S4.** List of PCR primers.

| **Primer name** | **Sequence (5'-3')** |
| --- | --- |
| GF100472F | CTGCTATACACATTTGTACC |
| GF100472R | CCTGTCTAATGTCAATATCC |
| rs339392F | GATGTTGCCCAGTCCAGTCT |
| rs339392R | CCAAAAAGTAGGAGGCTGGTT |
| rs2230199F | CAAGAATAATGGGCAGGCAAG |
| rs2230199R | GTCTTGTCTGTCTGGATGAAG |
| rs428453F | CCACAGCCCAACCTAGAAAA |
| rs428453R | TCCCACAGTTCCTGGTAACAA |
| rs344550F | TTTTTGTCCCCATGACCTTC |
| rs344550R | GGGCCAATTACTGTCACTCC |
| rs379527F | CCTGTATCTTTACCTTAACAGCATTG |
| rs379527R | GAGACGGGGTTTCATCATGT |
