# Supplementary material for: Functional Variant in Complement C3 Gene Promoter and Genetic Susceptibility to Temporal Lobe Epilepsy and Febrile Seizures
Source: PLoS One. 2010 Sep 16;5(9):e12740. doi: 10.1371/journal.pone.0012740 (PMC2940893; doi:10.1371/journal.pone.0012740)
Supplement: Table S5 — Linkage disequilibrium (LD) data. (0.04 MB DOC) [file pone.0012740.s005.doc]

**Table S5.** Linkage disequilibrium (LD) data. LD was calculated as D’ values for all single nucleotide polymorphism (SNP) pairs in healthy controls and in patients with mesial temporal lobe epilepsy (MTLE).

|  | D' value for polymorphism pair combinations in healthy controls | | | | | |
| --- | --- | --- | --- | --- | --- | --- |
|  | GF100472 | rs339392 | rs2230199 | rs428453 | rs344550 | rs379527 |
| GF100472 |  |  |  |  |  |  |
| rs339392 | 0.881 |  |  |  |  |  |
| rs2230199 | 0.784 | 0.672 |  |  |  |  |
| rs428453 | 0.388 | 0.161 | 0.233 |  |  |  |
| rs344550 | 0.170 | 0.059 | 0.16 | 0.079 |  |  |
| rs379527 | 0.196 | 0.034 | 0.041 | 0.05 | 0.839 |  |
|  | D' value for polymorphism pair combinations in MTLE patients | | | | | |
|  | GF100472 | rs339392 | rs2230199 | rs428453 | rs344550 | rs379527 |
| GF100472 |  |  |  |  |  |  |
| rs339392 | 0.999 |  |  |  |  |  |
| rs2230199 | 0.843 | 0.509 |  |  |  |  |
| rs428453 | 0.252 | 0.163 | 0.459 |  |  |  |
| rs344550 | 0.243 | 0.108 | 0.353 | 0.047 |  |  |
| rs379527 | 0.230 | 0.1 | 0.192 | 0.007 | 0.806 |  |
